# In-Home Cardiovascular Monitoring System for Heart Failure: Comparative Study

**DOI:** 10.2196/12419

**Published:** 2019-01-18

**Authors:** Nicholas J Conn, Karl Q Schwarz, David A Borkholder

**Affiliations:** 1 Microsystems Engineering Rochester Institute of Technology Rochester, NY United States; 2 University of Rochester Medical Center School of Medicine and Dentistry University of Rochester Rochester, NY United States

**Keywords:** ballistocardiogram, BCG, blood pressure, ECG, electrocardiogram, heart failure, Internet of Things, IoT, photoplethysmogram, PPG, remote monitoring, SpO2, stroke volume

## Abstract

**Background:**

There is a pressing need to reduce the hospitalization rate of heart failure patients to limit rising health care costs and improve outcomes. Tracking physiologic changes to detect early deterioration in the home has the potential to reduce hospitalization rates through early intervention. However, classical approaches to in-home monitoring have had limited success, with patient adherence cited as a major barrier. This work presents a toilet seat–based cardiovascular monitoring system that has the potential to address low patient adherence as it does not require any change in habit or behavior.

**Objective:**

The objective of this work was to demonstrate that a toilet seat–based cardiovascular monitoring system with an integrated electrocardiogram, ballistocardiogram, and photoplethysmogram is capable of clinical-grade measurements of systolic and diastolic blood pressure, stroke volume, and peripheral blood oxygenation.

**Methods:**

The toilet seat–based estimates of blood pressure and peripheral blood oxygenation were compared to a hospital-grade vital signs monitor for 18 subjects over an 8-week period. The estimated stroke volume was validated on 38 normative subjects and 111 subjects undergoing a standard echocardiogram at a hospital clinic for any underlying condition, including heart failure.

**Results:**

Clinical grade accuracy was achieved for all of the seat measurements when compared to their respective gold standards. The accuracy of diastolic blood pressure and systolic blood pressure is 1.2 (SD 6.0) mm Hg (N=112) and –2.7 (SD 6.6) mm Hg (N=89), respectively. Stroke volume has an accuracy of –2.5 (SD 15.5) mL (N=149) compared to an echocardiogram gold standard. Peripheral blood oxygenation had an RMS error of 2.3% (N=91).

**Conclusions:**

A toilet seat–based cardiovascular monitoring system has been successfully demonstrated with blood pressure, stroke volume, and blood oxygenation accuracy consistent with gold standard measures. This system will be uniquely positioned to capture trend data in the home that has been previously unattainable. Demonstration of the clinical benefit of the technology requires additional algorithm development and future clinical trials, including those targeting a reduction in heart failure hospitalizations.

## Introduction

### The Burden of Heart Failure

In-home monitoring technologies have the potential to transform the health care system by enabling the transition from reactive care to proactive and preventive care. This is especially important for cardiovascular disease (CVD), the leading cause of death worldwide. Heart failure (HF), a type of CVD characterized by a weakened heart muscle, impacts approximately 6.5 million Americans with over 960,000 new cases each year [1]. HF costs the United States an estimated $30.7 billion annually and is expected to increase 127% to $69.7 billion by 2030 [2]. With approximately 80% of the total cost associated with HF due to hospitalization [1], there is an opportunity to reduce the cost of HF by lowering hospitalization rates.

To contain costs, the Centers for Medicare and Medicaid Services is both penalizing hospitals with excess readmissions and moving to Bundled Payments for Care Improvement [3,4]. Despite increasing penalties [5], readmission rates remain high for HF, with over 20% of patients readmitted within 30 days and up to 50% by 6 months [6]. To successfully reduce readmissions, early detection of deterioration and subsequent intervention is required. Since patient awareness of symptomatology often lags behind deterioration, successfully tracking physiologic changes in the home is a critical component of an early intervention strategy.

### In-Home Monitoring to Reduce Heart Failure Hospitalization Rates

Current models for reducing HF hospitalizations through in-home monitoring have had mixed success due to delays in the analysis of potentially important clinical data [7], studies with insufficient power for drawing conclusions [8], and low adherence [9,10]. As an example, the Telemonitoring to Improve Heart Failure Outcomes trial for automated telemonitoring did not show a reduction in hospitalizations, due in part to low adherence [9]. In this large-scale study involving 826 subjects with telemonitoring, only 55% of the patients were using the system at the end of the trial. Similar results were found in the Baroreflex Activation Therapy for Heart Failure (BEAT-HF) study, where adherence was cited as a critical factor for not showing any change in HF hospitalizations with in-home monitoring of the electrocardiogram (ECG), weight, and blood pressure (BP) [10]. In this study, the subsection of the patients who had better adherence to monitoring had a significantly lower rate of hospital readmissions [11]. The BEAT-HF investigators stated “there remain difficulties in getting heart failure patients even to perform basic aspects of self-care, such as daily weight and BP monitoring” [11].

In contrast to these studies, the CardioMEMS Heart Sensor Allows Monitoring of Pressure to Improve Outcomes in NYHA Class III Heart Failure Patients (CHAMPION) trial demonstrated a 37% reduction in class III HF hospitalizations, where pulmonary artery pressures were measured daily through an implantable device (CardioMEMS) incorporating a patient-initiated data transfer using a bed-based system [12]. In comparison with prior studies that did not show a reduction in hospitalization, only 1.5% of the treatment group was noncompliant. In the CHAMPION Trial, the high level of adherence was due to the patient selection criteria, a preprocedure monitoring agreement, and the use of a nurse telephone intervention system [13]. In addition to high levels of adherence, the success of the CHAMPION trial can be attributed to the robust and clinically relevant measurements captured by the CardioMEMS device [12].

To have successful in-home monitoring with a nonimplantable system that is applicable to the broader patient population, a novel approach must be taken to bypass adherence issues. Furthermore, such a system must provide a sufficiently diverse and relevant set of measurements to practitioners that enable early detection of deterioration and intervention, as the epidemiology of HF is extremely complex.

### Measurements for Monitoring Heart Failure

HF occurs when the heart muscle is weakened and unable to maintain sufficient blood flow to meet the body’s needs. Consistent monitoring of BP is critical throughout the entire management and treatment of HF [4], as optimal BP control is a primary goal for HF [14]. In part, this is because lower systolic BP is associated with increase readmission and mortality rates [15,16], and uncorrelated high systolic BP typically precipitates acute decompensation [14]. Furthermore, according to the Framingham Study, early and continuous control of elevated BP appears to be the primary method for preventing chronic HF in the general population, with pulse and systolic pressure associated with significant risk for HF [17,18].

As HF is characterized by poor cardiac performance, cardiac output (CO) is an important component in diagnosis and management of HF [19]. CO is defined as the product of stroke volume (SV) and heart rate (HR) and is typically measured using an echocardiogram, a cardiac magnetic resonance imaging (MRI), or catheterization. In acute HF, the myocardium is unable to maintain sufficient CO and if untreated leads to chronic HF and death [20]. During diuretic treatment, CO and SV must not be significantly reduced and therefore must be monitored whenever possible to ensure optimal treatment [21]. Currently there is no in-home solution for remotely and accurately monitoring CO and SV. As such, the benefits and predictive value of monitoring CO and SV on a daily basis remain unproven.

The epidemiology of HF is complex, and a limited set of measurements is often insufficient for making clinical decisions. Peripheral oxygenation saturation (SpO_2_) has value as a supporting measure that can be used to determine the best course for treatment during the initial diagnosis, acute HF, and acute decompensation [20,22]. During acute decompensation, patients presenting with hypoxemia (oxygen saturation <90%) warrant hospitalization and require daily monitoring of oxygen saturation and other vital signs until stabilization [23].

The objective of this study was to demonstrate that a toilet seat–based cardiovascular monitoring system is capable of measuring systolic and diastolic blood pressures, stroke volume, and blood oxygenation.

## Methods

### A Toilet Seat–Based Cardiovascular Monitoring System for In-Home Monitoring

A toilet seat–based cardiovascular monitoring system can be integrated into a subject’s natural daily routine with no change in habit, enabling measurements to be taken at one or more times each day. Issues with subject preparation and subject error are greatly reduced, since skin contact is automatic and highly repeatable each use. While a toilet seat–based monitoring system is intermittent in nature, ensured adherence will enable long-term daily trend monitoring of parameters that do not need to be captured continuously, such as BP.

**Figure 1:**
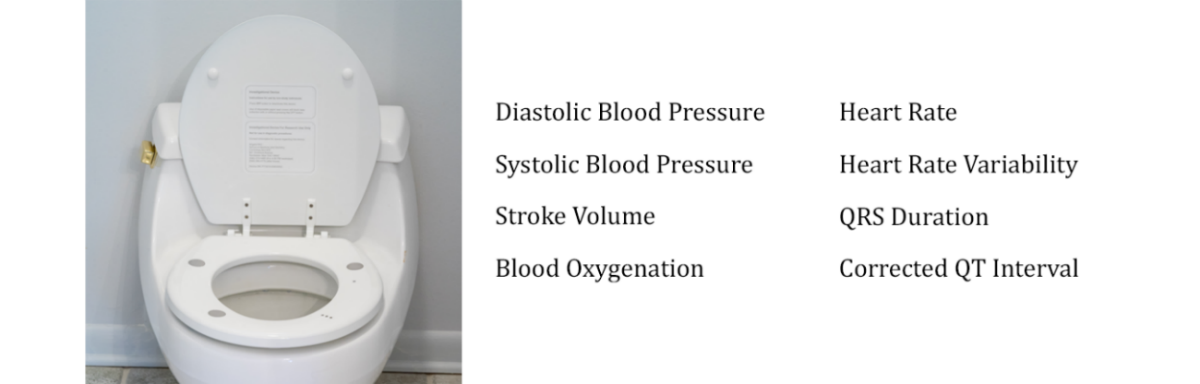
A toilet seat–based cardiovascular monitoring system (left) is integrated into an individual’s daily routine without requiring any change in habit, thereby addressing patient adherence. The system captures a comprehensive set of clinically relevant measurements automatically (right).

This work demonstrates that a toilet seat–based monitoring system (Figure 1) is capable of accurately capturing the following clinically relevant parameters: BP, SV, and blood oxygenation. The single-lead ECG measured from the seat has been previously correlated to the 12-lead ECG and validated against standard lead II for HR, heart rate variability, QRS duration, and the corrected QT interval [24] and will not be discussed herein. This set of measurements, gathered from a single device, provides a broad view of a patient’s cardiovascular health.

### An Integrated System for Cardiovascular Monitoring

**Figure 2:**
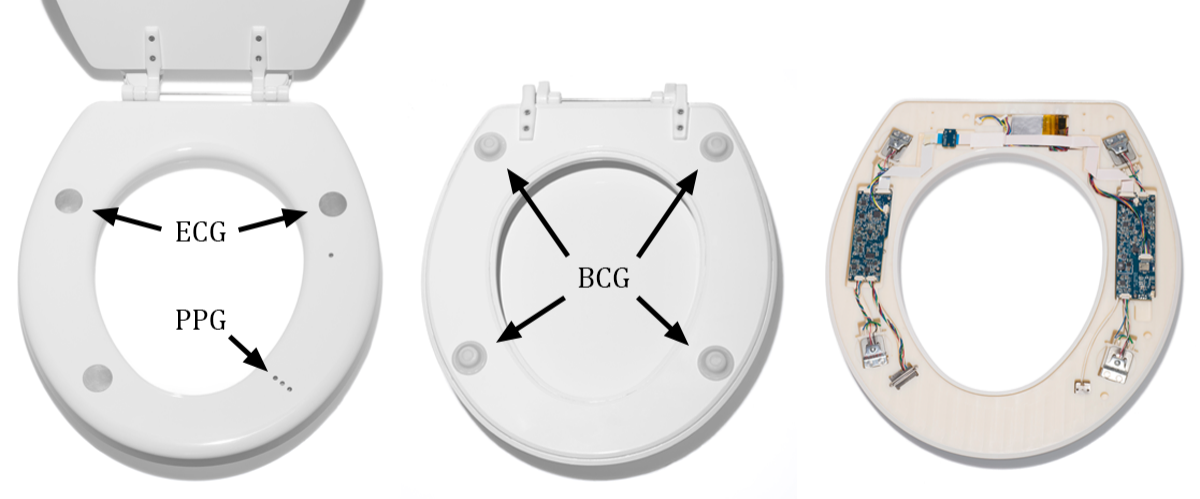
The toilet seat–based cardiovascular monitoring system is completely self-contained, battery-powered, wireless, and cleanable with all sensors and electronics instrumentation integrated inside of the seat. It can measure the electrocardiogram (ECG), photoplethysmogram (PPG), and the ballistocardiogram (BCG).

The proposed cardiovascular monitoring system installs directly on a standard toilet, is battery powered, wireless, waterproof, and requires no additional connections or user interaction (Figure 2). This monitoring system unobtrusively captures cardiovascular data automatically whenever the user sits on the toilet. Requiring no direct user actions for measurement, patient adherence is enhanced. The seat incorporates a single-lead ECG for measuring the electrical activity of the heart and as a reference for ensemble averaging [24], a ballistocardiogram (BCG) for measuring the mechanical forces associated with the cardiac cycle, and a photoplethysmogram (PPG) for measuring SpO_2_ and pulse transit time (PTT) (Figure 2).

While many methods (eg, echocardiography) are available to clinicians for measuring ventricular performance (eg, CO), all require costly dedicated equipment that is generally deployed in a formal medical setting. These methods require expert technicians and interpretation that can be subject to reviewer bias or geometric errors. Because of these limitations, none of these techniques can be used for day-to-day monitoring of cardiac function or be incorporated into nonimplantable, in-home, medical diagnostic devices. The BCG fills this gap and enables devices to be created that are capable of monitoring cardiac function in the home in an inexpensive way [25-27].

**Figure 3:**
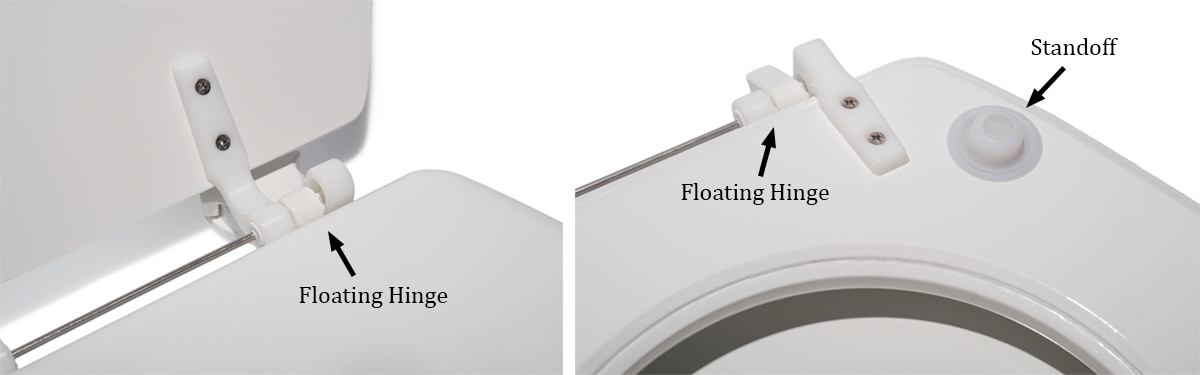
A floating hinge ensures that the weight on the seat is completely captured by the load cells under each standoff rather than having a portion of it carried by the hinge. This is a requirement for accurate ballistocardiogram monitoring on a toilet seat.

The force present on the seat is measured through 4 independent load cells underneath 4 standoffs placed on the bottom of the seat (Figures 2 and 3). To accurately measure the small forces that correspond to the BCG on a toilet seat, a floating hinge (Figure 3) is required to ensure that all of the load is captured by the load cells [28].

**Figure 4:**
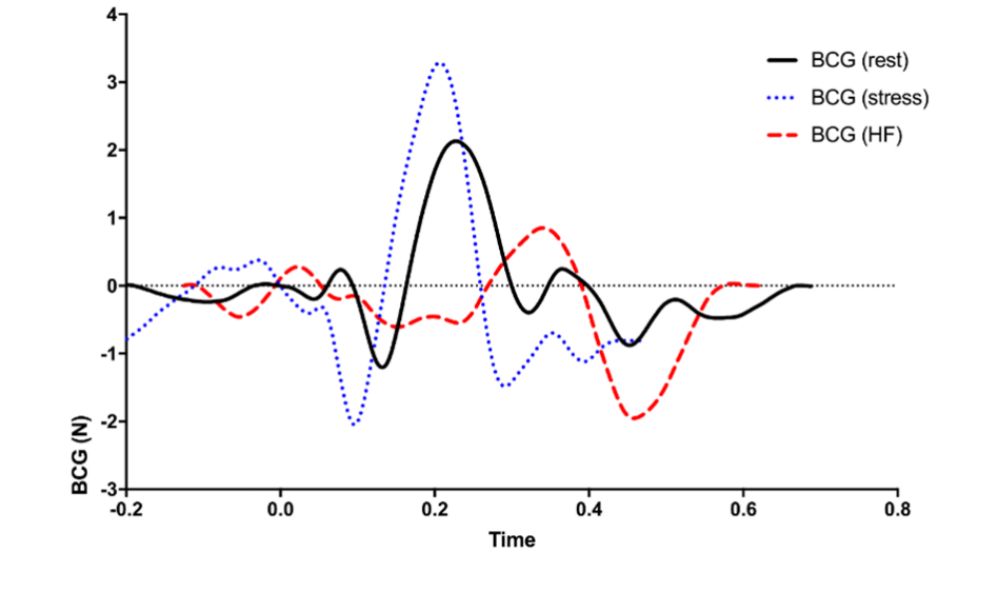
The ballistocardiogram (BCG) amplitude and timing vary greatly based on the cardiovascular state. Heart failure (HF) BCG waveforms have a much smaller amplitude when compared with the normal BCG waveform at rest and poststress.

The BCG waveform has been shown to correlate with the pre-ejection period and CO [25,27,29]. To demonstrate the correlation between BCG amplitude and cardiac function, differences in the ensemble averaged BCG waveforms measured from the seat for a typical HF subject and for a typical normative subject at both rest and post-stress are shown in Figure 4. In combination with the ECG, the BCG is used to estimate SV and calculate the starting point for the pulse transit time for BP estimation.

The PPG is an optical measure of local blood volume [30]. When captured at 2 wavelengths (eg, red and infrared), SpO_2_ can be estimated [30]. The ratio between the waveform amplitudes from each wavelength is used to calculate an R-value, which can then be converted to SpO_2_ through a device-specific calibration curve (R-curve) [30,31]. Typically, SpO_2_ is measured on the finger or the earlobe, where intersubject variability in the local tissue is low, allowing for a universal R-curve to be used across the entire population. When measuring SpO_2_ from the back of the thigh, as is the case with the seat, a single point calibration is required for each subject due to the variability in local tissue. Additionally, the single point calibration for each subject will mitigate the sensitivity of the SpO_2_ estimate to skin pigment, which becomes a potential source of error during hypoxia (<70%) [32].

**Figure 5:**
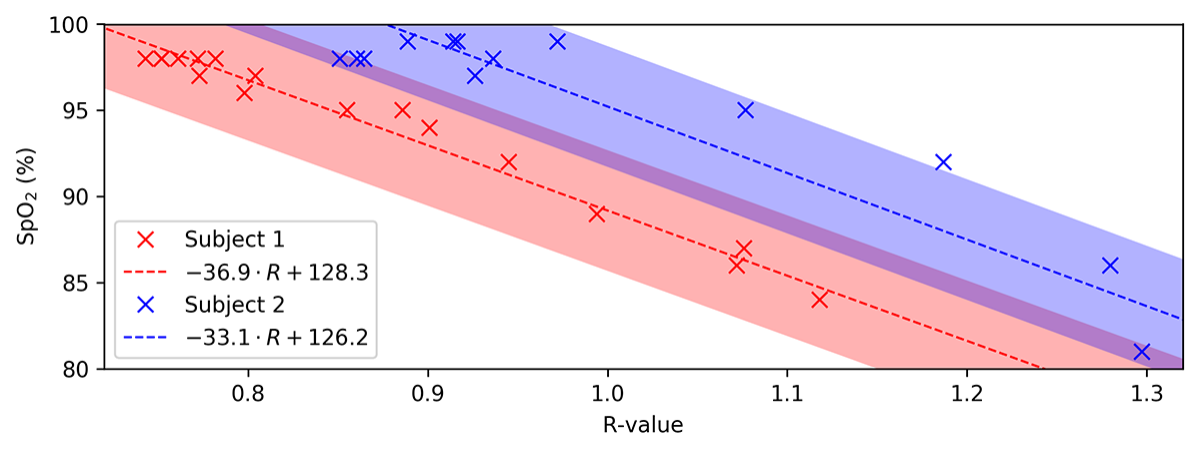
When characterizing the R-value for SpO_2_ (peripheral oxygen saturation) estimation on the seat with controlled desaturation testing, the R-curve slope matches literature and is the same across subjects. A different offset necessitates the use of a per-subject calibration for absolute SpO_2_ estimation. The shaded regions represent the acceptable level of error around the best fit line according to the ISO standard for pulse oximetry.

A controlled desaturation test was performed on 2 subjects of varying weight and body type to demonstrate the difference in R-curves between subjects as measured on the seat. Each subject slowly reduced their SpO_2_ to approximately 80% while data were captured on the seat. An R-value was calculated from the seat data for various saturation levels as measured by a gold standard pulse oximeter, which was taken from the finger with a hospital grade vital signs monitor (ProCare 400 Vital Signs Monitor, General Electric Company). This was used to generate an R-curve for each subject (Figure 5), where the shaded region is the potential error of the hospital grade vital signs monitor.

The slope of each subject’s R-curve (–36.9 and –33.1) varies by 11% and matches that expected in literature (–33.3) [31]. While the variation in slopes is small, the difference in offset between the R-curves was significant. This indicates that while the seat is capable of measuring relative changes in SpO_2_ without a per-subject calibration, a single point calibration is required for providing an absolute measure of SpO_2_.

One of the key benefits of the proposed system is the ability to use a combination of the ECG, BCG, and PPG to extract meaningful parameters such as BP. Literature shows that it is possible to estimate BP from pulse wave velocity (PWV) [33-36], which is the speed at which the pressure wave propagates through the arterial system. An aortic PWV can be measured from the seat using an estimate of the subject’s aortic length and the PTT. The aortic PTT is defined as the time it takes for the pressure wave to transit the aorta. The seat calculates this by determining the time interval between the BCG feature that relates to ejection and the appropriate peripheral PPG wave feature.

There are two key differences between the seat and the majority of other PWV-based estimates of BP that allow for a more accurate and robust determination of BP. Many examples in literature use the pulse arrival time instead of the PTT, which uses the ECG as the proximal timing point for calculating the time of propagation [37-39] and includes a portion of the pre-ejection period in the overall time estimate. This results in an inaccurate estimate of transit time as the ECG timing is a poor surrogate for ejection timing [36,37]. Additionally, the peripheral timing point for the seat-based PTT is measured on the back of the thigh, which is in close proximity to the end of the aorta. This is in contrast to other peripheral measurement sites, such as the finger or foot. In these cases, the PTT, and the subsequent PWV measure, is not dominated by the aorta resulting in a less robust correlation to BP.

### Human Subject Testing

Diastolic BP, systolic BP, SV, and SpO_2_ are validated with human subject data obtained from studies at the Rochester Institute of Technology and the University of Rochester Medical Center. Studies were performed under informed consent and used protocols approved by each institution’s Institutional Review Board for Protection of Human Subjects. General exclusion criteria for all of the studies include subjects who are less than 18 years of age, pregnant, weigh more than 180 kg, cannot follow instructions in English, or have mechanical circulatory support or impaired cognitive or functional status. Each controlled study compares the capabilities of the seat to a clinical grade gold standard, quantitatively comparing the accuracy of each measure.

For each of the following studies, recordings were captured in a lab or clinical setting. Subjects were instructed not to urinate or defecate, not to talk, and to sit as they normally would in their home when recordings were captured. No other instructions were given. As urination and defecation can shift BP, SV, and HR, it is not the intention of this work to analyze the physiologic changes during urination or defecation but rather to track daily trends at steady state. In future in-home studies, algorithms will be developed to identify and reject periods of urination and defecation through classification of motion artifacts and the physiologic shifts associated with this change in state.

Prior to parameter estimation and feature extraction, each of the signals undergoes a continuous signal quality check where entire recordings may be rejected, and beats with abnormal intervals are removed, since changes in the diastolic duration and ventricular filling can significantly shift beat-by-beat BP and SV, as described in Conn et al [24,28]. The demographic information for the remaining subjects in each cohort, grouped by measurement, are shown in Table 1.

**Table 1:** Demographic information for the cohorts used to validate each of the seat measures. These statistics only include data that have passed the automated signal quality check.

Cohort	Male (n)	Age (years)	Weight (kg)	Height (cm)	Body mass index (kg/m^2^)
Diastolic BP^a^ (n=12)	9	22.5 (2.8)	76.1 (13.5)	173.6 (9.3)	25.2 (3.6)
Systolic BP (n=11)	9	22.6 (2.9)	76.7 (13.9)	175.2 (7.9)	24.9 (3.5)
SV^b^ Normative (n=38)	22	24.5 (5.6)	73.3 (21.1)	171.8 (7.6)	24.7 (6.2)
SV In-Clinic (n=111)	59	55.7 (16.4)	81.3 (19.5)	170.6 (10.3)	27.8 (5.6)
SpO_2_^c^ (n=11)	10	22.5 (2.9)	77.6 (12.9)	173.9 (9.5)	25.6 (3.4)

^a^BP: blood pressure.

^b^SV: stroke volume.

^c^SpO_2_: peripheral oxygen saturation.

Systolic and diastolic BP was validated on 12 and 11 normative (healthy) subjects, respectively, with no history of heart disease, chronic obstructive pulmonary disease, diabetes, or peripheral vascular disease. A single measurement session was used for calibrating the blood pressure estimator. For each subject, a maximum of 5 recordings was taken per week for a total of 8 weeks. A maximum of 2 recordings was allowed per day with a minimum of 4 hours separating subsequent recordings.

During in-home use of the seat, it cannot be guaranteed that the subject will follow the recommendations of the American Heart Association, where BP should be measured after the subject has sat at rest for at least 5 minutes with their back supported by a chair [40]. To ensure that the seat accurately measures the subject’s BP during typical use, the gold standard measure of brachial BP was captured from a hospital-grade vital signs monitor (ProCare 400 Vital Signs Monitor, General Electric Company) before, during, and after every seat recording, with the average value compared to the seat estimate. As the PTT measured from the seat is dominated by the aorta, the resulting BP estimate is most related to central BP. While the differences between central pressures and brachial pressures are variable between individuals [41], the shifts in pressure will track together within a single subject, allowing for a comparison to brachial BP over time. This potential source of error is mitigated by performing a per-subject calibration of the PWV model to brachial pressures.

SV was validated on 38 normative subjects and 111 patients undergoing a standard echocardiogram at the University of Rochester Medical Center for any underlying condition, including HF. For each of the subjects in this cohort, a reference measure of SV was gathered at rest while the subject was supine using an echocardiogram (Vivid i, General Electric Company). SV was calculated from the velocity time interval at the left ventricular outflow tract using Doppler mode with all estimations performed by a single cardiologist to eliminate interobserver variability. The seat data were gathered during the same session while at rest and compared to the echocardiogram measure of SV.

SpO_2_ was validated on 11 normative subjects with no history of heart disease, chronic obstructive pulmonary disease, diabetes, or peripheral vascular disease. A single calibration session was used to calculate the subject-specific R-curve offset. For each subject, a maximum of 5 recordings was taken per week for a total of 8 weeks. A maximum of 2 recordings was allowed per day, with a minimum of 4 hours separating subsequent recordings. A gold standard measure of SpO_2_ was captured from a hospital-grade vital signs monitor (ProCare 400 Vital Signs Monitor) before and after every recording. The average SpO_2_ value was used to determine the accuracy of the seat estimate.

## Results

### Blood Pressure Estimates Robustly Correlate to Gold Standard

**Figure 6:**
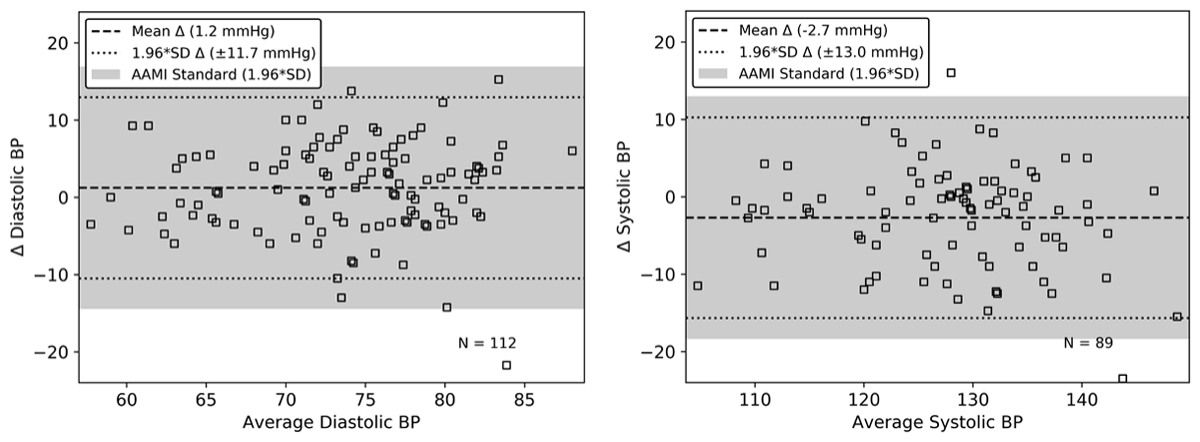
The toilet seated–based cardiovascular monitoring system has been shown to accurately measure blood pressure over an 8-week period. Both the diastolic (left) and systolic (right) blood pressure (BP) estimates from the seat exceed the accuracy required by the Association for the Advancement of Medical Instrumentation (AAMI) standard converted to a limits of agreement (shaded regions).

The Bland-Altman plots in Figure 6 demonstrate that diastolic (left) and systolic (right) BP estimates compare favorably to a clinical gold standard. Additional recordings were automatically rejected for systolic BP (N=89) compared to diastolic BP (N=112) because signal quality requirements are more stringent for systolic BP. The resulting error for the diastolic BP is 1.2 (SD 6.0) mm Hg and the resulting error for the systolic BP is –2.7 (SD 6.6) mm Hg. These results exceed the Association for the Advancement of Medical Instrumentation (AAMI) standards, which require all measurements across every subject to have an accuracy lower than ±5 (SD 8) mm Hg [42]. For visualization on the Bland-Altman plots in Figure 6, the required SD has been converted to a limits of agreement (SD multiplied by 1.96) and is shown as a shaded region around the mean. These data demonstrate that the seat is capable of tracking shifts in blood pressure over time without recalibration.

When the estimation error is stratified by body mass index (BMI), the seat’s estimate of BP slightly overestimates diastolic BP and underestimates systolic compared to the gold standard for larger BMIs. This trend is more significant for the systolic BP estimate. The error in estimating both systolic and diastolic BP stratified by BMI for this cohort is shown in Table 2.

### Accurate Stroke Volume Estimation Across a Large and Diverse Population

**Figure 7:**
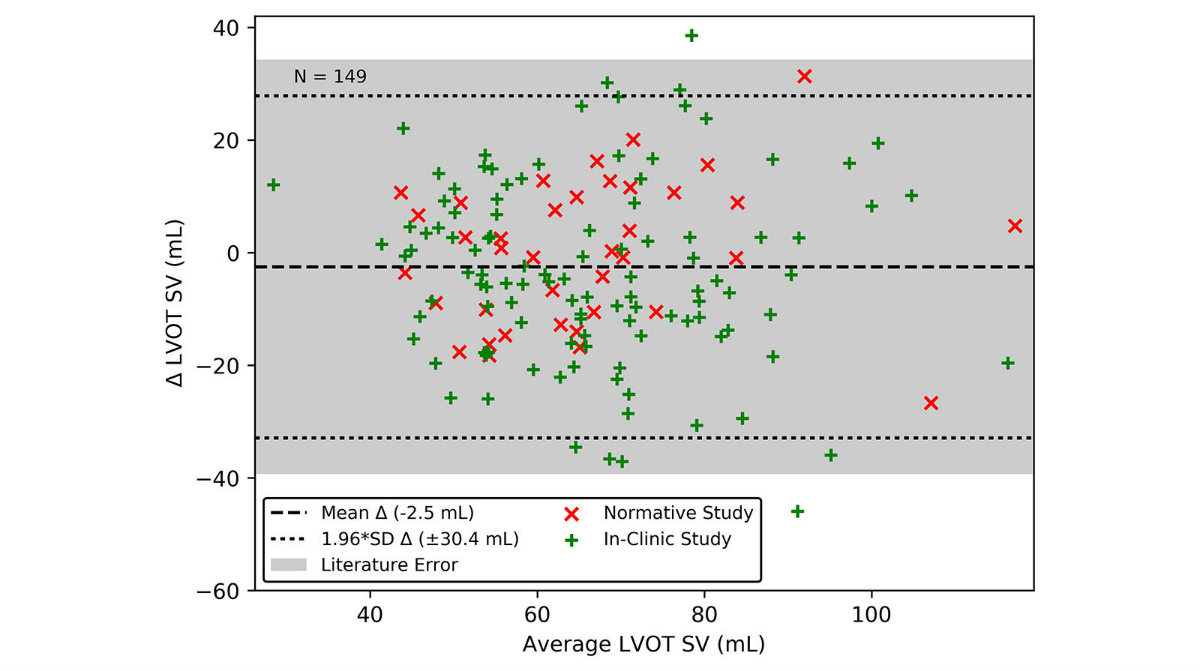
The seat estimate of stroke volume (SV) strongly correlates to the echocardiography measure of SV measured from the left ventricular outflow tract (LVOT). In comparison, the literature shows that the echocardiogram SV measure has a limits of agreement of 35.2 mL (shaded region) compared to an arterial gold standard.

The seat estimate of SV is compared to the echocardiogram in a Bland-Altman plot (Figure 7). The mean and SD of the heart rate across all subjects in this cohort is 74.3 (SD 12.4) bpm with a range of 47.8 bpm to 113.2 bpm. Literature indicates that the limits of agreement (1.96 SD) for an echocardiogram Doppler-based measure of SV is 35.2 mL when calculated from the velocity time integral [43,44]. This is shown as a shaded region in Figure 7. Similarly, the limits of agreement for SV calculated using a cardiac MRI compared to thermodilution is 22 mL [45]. These compare favorably with the seat estimate of SV across a diverse population with limits of agreement of 30.4 mL when compared to an echocardiogram (Figure 7). When stratified by BMI, the limits of agreement are as follows: 15.3 mL (N=4) for a BMI less than 18.5, 26.6 mL (N=56) for a BMI between 18.5 and 25, 34.6 mL (N=56) for a BMI between 25 and 30, and 29.5 mL (N=33) for a BMI over 30. This indicates that the ability of the seat to estimate SV is not significantly impacted by BMI.

### Consistent Estimation of Peripheral Blood Oxygenation

**Figure 8:**
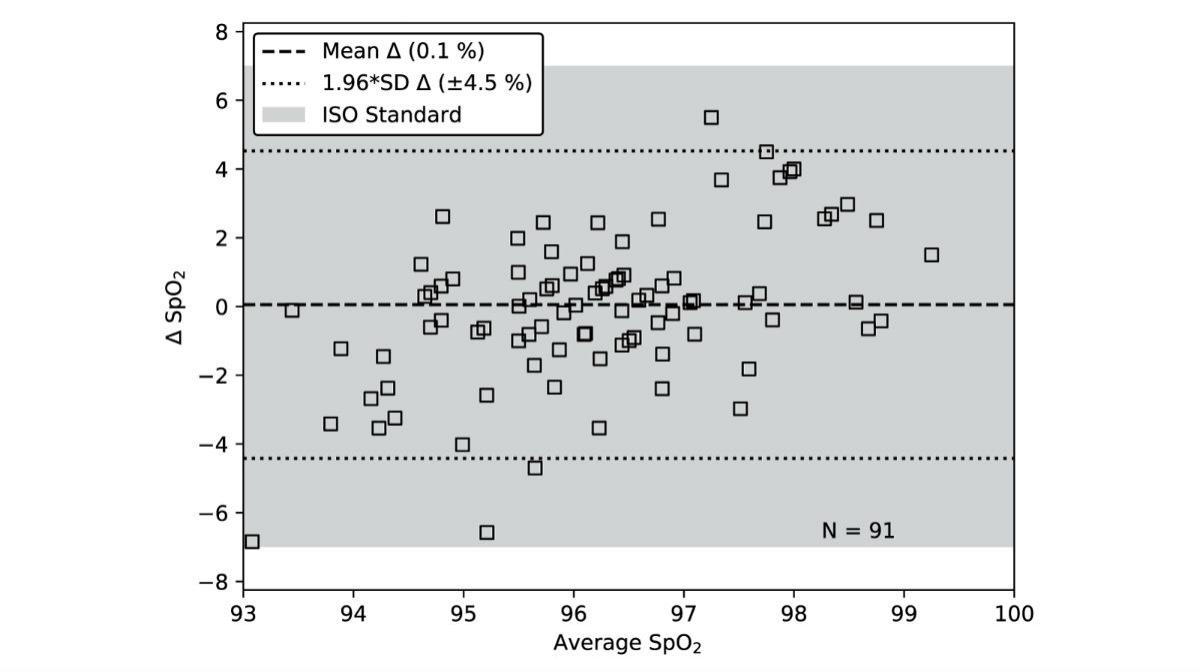
The limits of agreement for SpO_2_ (peripheral oxygen saturation) is 4.5% with an A_RMS_ (root mean square error) of 2.3%. This exceeds the accuracy required by the ISO standard for SpO_2_ where A_RMS, MAX_ is 3.5%, which equates to a limits of agreement of 6.9% (shaded region).

The Bland-Altman plot in Figure 8 shows the accuracy of the seat’s estimate of SpO_2_ compared to the gold standard for 91 data points collected from 11 subjects over a period of 8 weeks. The resulting root mean square error (A_RMS_) of the seat estimate of SpO_2_ compared to the gold standard is 2.3%. This exceeds the accuracy required by the ISO standard for SpO_2_ (A_RMS,MAX_=3.5%) [46]. Assuming a zero mean for visualization purposes, the required A_RMS,MAX_ can be converted to a limits of agreement (shaded region in Figure 8) by multiplying by 1.96.

**Table 2:** Error in blood pressure estimation for this cohort stratified by body mass index at the time of calibration (no subjects in this cohort had a body mass index lower than 18.5 kg/m^2^).

BMI^a^, range	Diastolic (mm Hg)		Systolic (mm Hg)	
	n	mean (SD)	n	mean (SD)
18.5≤BMI<25.0	52	–0.1 (6.5)	49	–1.8 (7.1)
25.0≤BMI<30.0	50	2.0 (5.4)	34	–3.5 (5.8)
30.0≤BMI	10	3.6 (4.4)	6	–5.6 (5.3)

^a^BMI: body mass index; expressed in kg/m^2^.

## Discussion

### Principal Results

This work demonstrates that a toilet seat–based cardiovascular monitoring system can robustly measure systolic and diastolic BP, SV, and SpO_2_ compared to their respective clinical gold standards (Table 3). Results show that SV can be estimated absolutely with an accuracy comparable to the echocardiogram, which is the most commonly used method for measuring SV.

**Table 3:** Principal results for each of the seat measures compared to their respective clinical gold standards.

Metric	Diastolic BP^a^ (mm Hg; n=112)	Systolic BP (mm Hg; n=89)	SV^b^ (mL; n=149)	A_RMS_^c^ SpO_2_^d^ (%; n=91)
Error	1.2 (6.0)	–2.7 (6.6)	–2.5 (30.4)^e^	2.3
Target	±5.0 (8.0)	±5.0 (8.0)	±2.0 (35.2)^e^	3.5

^a^BP: blood pressure.

^b^SV: stroke volume.

^c^A_RMS_: root mean square error.

^d^SpO_2_: peripheral oxygen saturation.

^e^Error and target presented as limits of agreement (1.96*SD).

Both systolic and diastolic BP can be measured in a normative population over a period of 8 weeks using the ECG, BCG, and PPG with a per-subject calibration within the AAMI standards [42]. The seat is capable of measuring a subject’s SpO_2_ using a reflectance mode pulse oximeter positioned to make contact with the upper thigh with a single-point calibration for each subject. The single-lead ECG, HR, HRV, QRS duration, and corrected QT interval have been previously validated [24].

### Limitations and Future Work

One limitation of this work is that the seat-based system has not been used extensively in the home setting. In each of these studies, the seat was set up for automatic detection and data acquisition upon subject use with unattended data transmission to a cloud database. There was no use of lab or clinic infrastructure for gathering seat data, and the seat was deployed exactly as it would be in the home. While BP and SpO_2_ were measured over multiple weeks, the majority of subjects did not show significant shifts within this time frame. Another limitation of the work is that the limits of agreement for SV is similar to that of the echocardiogram. It is unknown whether the errors in the estimation of SV are due to the seat or the echocardiographic measure of the left ventricular outflow tract area. As such, future studies will compare the seat’s estimate of SV to a gold standard cardiac MRI.

Future work will initially focus on larger scale studies to validate BP and SpO_2_ across a broader population. As urination and defecation can shift BP, SV, and HR, detection algorithms will be developed and validated to remove these states from the analysis, ensuring that accurate daily trends at steady state are captured. Subject identification using biometrics to enable truly passive monitoring in a multiple person household will also be developed. Additionally, the possibility of removing the per-subject calibration required for BP and SpO_2_ estimation will be investigated through the incorporation of easily measured subject-specific information (eg, thigh thickness). Body weight measurements on the seat have not yet been verified. Future studies will investigate the ability of machine learning to estimate body weight based on posture and body type. This work and the aforementioned future studies will lead to a 2-phased clinical trial where an alert-based system for early detection of deterioration will be developed with in-home seat data and subsequently validated with the goal of demonstrating a reduction in HF hospitalization rates.

### Broad Impact

The toilet seat–based cardiovascular monitoring system has the potential fill a gap in patient monitoring by capturing trend data that has been previously unattainable. This system has the potential to address many of the challenges with in-home monitoring in a form factor that integrates into the daily routine of patients, bypassing barriers to adherence and providing a comprehensive and accurate set of clinically relevant measurements. In addition to in-home monitoring, a secondary use for this device includes monitoring of patients in the hospital. While BP and SpO_2_ are routinely monitored, daily measurements of SV and CO can be used to provide additional insights into the effectiveness of ongoing treatments.

Such a device may enable new approaches and capabilities in the diagnosis and treatment of cardiovascular disease, including but not limited to those with HF. After further demonstration of the measurement capabilities of the seat, this device will be uniquely positioned to advance an automated alert-based system that could be part of a broader interventional strategy in future clinical trials, with the goal of reducing HF hospitalizations. If successful, this strategy has the potential to reduce the burden of HF and cardiovascular disease on the health care industry as well as improve the quality of life for patients. Through the successful development, deployment, and integration with clinical practice, this device could facilitate the transition from a reactive to proactive-based approach to health care.

## Abbreviations

AAMI: Association for the Advancement of Medical Instrumentation
A_RMS_: root mean square error
BCG: ballistocardiogram
BEAT-HF: Baroreflex Activation Therapy for Heart Failure
BMI: body mass index
BP: blood pressure
CHAMPION: CardioMEMS Heart Sensor Allows Monitoring of Pressure to Improve Outcomes in NYHA Class III Heart Failure Patients
CO: cardiac output
CVD: cardiovascular disease
ECG: electrocardiogram
HF: heart failure
HR: heart rate
MRI: magnetic resonance imaging
PPG: photoplethysmogram
PWV: pulse wave velocity
PTT: pulse transit time
SpO_2_: peripheral oxygen saturation
SV: stroke volume

## References

[1] Benjamin EJ, Blaha MJ, Chiuve SE, Cushman M, Floyd J, Fornage M, Gillespie C, Isasi CR, Jiménez MC, Jordan LC, Judd SE, Lackland D, Lichtman JH, Lisabeth L, Liu S, Longenecker CT, Mackey RH, Matsushita K, Mozaffarian D, Mussolino ME, Nasir K, Neumar RW, Palaniappan L, Pandey DK, Thiagarajan RR, Reeves MJ, Ritchey M, Rodriguez CJ, Roth GA, Rosamond WD, Sasson C, Towfighi A, Tsao CW, Turner MB, Virani SS, Voeks JH, Willey JZ, Wilkins JT, Wu JH, Alger HM, Wong SS, Muntner P (2017). Heart disease and stroke statistics—2017 update: a report from the American Heart Association. Circulation.

[2] Heidenreich PA, Trogdon JG, Khavjou OA, Butler J, Dracup K, Ezekowitz MD, Finkelstein EA, Hong Y, Johnston SC, Khera A, Lloyd-Jones DM, Nelson SA, Nichol G, Orenstein D, Wilson PWF, Woo YJ (2011). Forecasting the future of cardiovascular disease in the United States: a policy statement from the American Heart Association. Circulation.

[3] (2018). Centers for Medicare & Medicaid Services.

[4] (2016). Centers for Medicare & Medicaid Services.

[5] McIlvennan CK, Eapen ZJ, Allen LA (2015). Hospital readmissions reduction program. Circulation.

[6] O'Connor CM (2017). High heart failure readmission rates: is it the health system's fault?. JACC Heart Fail.

[7] Anker SD, Koehler F, Abraham WT (2011). Telemedicine and remote management of patients with heart failure. Lancet.

[8] Koehler F, Winkler S, Schieber M, Sechtem U, Stangl K, Böhm M, Boll H, Baumann G, Honold M, Koehler K, Gelbrich G, Kirwan B, Anker SD (2011). Impact of remote telemedical management on mortality and hospitalizations in ambulatory patients with chronic heart failure: the telemedical interventional monitoring in heart failure study. Circulation.

[9] Chaudhry SI, Mattera JA, Curtis JP, Spertus JA, Herrin J, Lin Z, Phillips CO, Hodshon BV, Cooper LS, Krumholz HM (2010). Telemonitoring in patients with heart failure. N Engl J Med.

[10] Ong MK, Romano PS, Edgington S, Aronow HU, Auerbach AD, Black JT, Escarce JJ, Evangelista LS, Hanna B, Ganiats TG, Greenberg BH, Greenfield S, Kaplan SH, Kimchi A, Liu H, Lombardo D, Mangione CM, Sadeghi B, Sadeghi B, Sarrafzadeh M, Tong K, Fonarow GC, Better Effectiveness After Transition–Heart Failure (BEAT-HF) Research Group (2016). Effectiveness of remote patient monitoring after discharge of hospitalized patients with heart failure: the better effectiveness after transition—Heart Failure (BEAT-HF) randomized clinical trial. JAMA Intern Med.

[11] O'Riordan M (2015). Heartwire from Medscape.

[12] Abraham WT, Adamson PB, Bourge RC, Aaron MF, Costanzo MR, Stevenson LW, Strickland W, Neelagaru S, Raval N, Krueger S, Weiner S, Shavelle D, Jeffries B, Yadav JS (2011). Wireless pulmonary artery haemodynamic monitoring in chronic heart failure: a randomised controlled trial. Lancet.

[13] Rathman LD, Fiorini DM, Nissley KM, Kurtz KM, Small RS, Roberts JD (2016). Holding up both ends of the bargain: ambulatory hemodynamic monitoring using CardioMEMS. J Cardiac Fail.

[14] Yancy CW, Jessup M, Bozkurt B, Butler J, Casey DE, Drazner MH, Fonarow GC, Geraci SA, Horwich T, Januzzi JL, Johnson MR, Kasper EK, Levy WC, Masoudi FA, McBride PE, McMurray JJV, Mitchell JE, Peterson PN, Riegel B, Sam F, Stevenson LW, Tang WHW, Tsai EJ, Wilkoff BL (2013). 2013 ACCF/AHA guideline for the management of heart failure: a report of the American College of Cardiology Foundation/American Heart Association Task Force on Practice Guidelines. J Am Coll Cardiol.

[15] Gheorghiade M, Abraham WT, Albert NM, Greenberg BH, O'Connor CM, She L, Stough WG, Yancy CW, Young JB, Fonarow GC, OPTIMIZE-HF Investigators and Coordinators (2006). Systolic blood pressure at admission, clinical characteristics, and outcomes in patients hospitalized with acute heart failure. JAMA.

[16] Chin MH, Goldman L (1997). Correlates of early hospital readmission or death in patients with congestive heart failure. Am J Cardiol.

[17] Kannel WB, Castelli WP, McNamara PM, McKee PA, Feinleib M (1972). Role of blood pressure in the development of congestive heart failure. The Framingham study. N Engl J Med.

[18] Haider AW, Larson MG, Franklin SS, Levy D, Framingham Heart Study (2003). Systolic blood pressure, diastolic blood pressure, and pulse pressure as predictors of risk for congestive heart failure in the Framingham Heart Study. Ann Intern Med.

[19] Mann D, Zipes D, Libby P, Bonow R (1988). Braunwald's Heart Disease: A Textbook of Cardiovascular Medicine, Third Edition.

[20] Nieminen MS, Böhm M, Cowie MR, Drexler H, Filippatos GS, Jondeau G, Hasin Y, Lopez-Sendon J, Mebazaa A, Metra M, Rhodes A, Swedberg K, Priori SG, Garcia MAA, Blanc J, Budaj A, Cowie MR, Dean V, Deckers J, Burgos EF, Lekakis J, Lindahl B, Mazzotta G, Morais J, Oto A, Smiseth OA, Garcia MAA, Dickstein K, Albuquerque A, Conthe P, Crespo-Leiro M, Ferrari R, Follath F, Gavazzi A, Janssens U, Komajda M, Morais J, Moreno R, Singer M, Singh S, Tendera M, Thygesen K, ESC Committe for Practice Guideline (CPG) (2005). Executive summary of the guidelines on the diagnosis and treatment of acute heart failure: the Task Force on Acute Heart Failure of the European Society of Cardiology. Eur Heart J.

[21] Remme WJ, Swedberg K, Task Force for the Diagnosis and Treatment of Chronic Heart Failure‚ European Society of Cardiology (2001). Guidelines for the diagnosis and treatment of chronic heart failure. Eur Heart J.

[22] Mebazaa A, Yilmaz MB, Levy P, Ponikowski P, Peacock WF, Laribi S, Ristic AD, Lambrinou E, Masip J, Riley JP, McDonagh T, Mueller C, deFilippi C, Harjola V, Thiele H, Piepoli MF, Metra M, Maggioni A, McMurray J, Dickstein K, Damman K, Seferovic PM, Ruschitzka F, Leite-Moreira AF, Bellou A, Anker SD, Filippatos G (2015). Recommendations on pre-hospital & early hospital management of acute heart failure: a consensus paper from the Heart Failure Association of the European Society of Cardiology, the European Society of Emergency Medicine and the Society of Academic Emergency Medicine. Eur J Heart Fail.

[23] Joseph SM, Cedars AM, Ewald GA, Geltman EM, Mann DL (2009). Acute decompensated heart failure: contemporary medical management. Tex Heart Inst J.

[24] Conn NJ, Schwarz KQ, Borkholder DA (2018). Nontraditional electrocardiogram and algorithms for inconspicuous in-home monitoring: comparative study. JMIR Mhealth Uhealth.

[25] Inan OT, Etemadi M, Paloma A, Giovangrandi L, Kovacs GTA (2009). Non-invasive cardiac output trending during exercise recovery on a bathroom-scale-based ballistocardiograph. Physiol Meas.

[26] Inan OT, Etemadi M, Wiard RM, Giovangrandi L, Kovacs GTA (2009). Robust ballistocardiogram acquisition for home monitoring. Physiol Meas.

[27] Giovangrandi L, Inan OT, Wiard RM, Etemadi M, Kovacs GTA (2011). Ballistocardiography—a method worth revisiting. Conf Proc IEEE Eng Med Biol Soc.

[28] Conn N (2016). Robust Algorithms for Unattended Monitoring of Cardiovascular Health [Dissertation].

[29] Inan OT, Etemadi M, Wiard RM, Kovacs GTA, Giovangrandi L (2008). Non-invasive measurement of Valsalva-induced hemodynamic changes on a bathroom scale ballistocardiograph. Conf Proc IEEE Eng Med Biol Soc.

[30] Webster J (1997). Design of Pulse Oximeters.

[31] Azmal G (2006). Continuous measurement of oxygen saturation level using photoplethysmography signal.

[32] Bickler PE, Feiner JR, Severinghaus JW (2005). Effects of skin pigmentation on pulse oximeter accuracy at low saturation. Anesthesiology.

[33] Lillie JS, Liberson AS, Mix D, Schwarz KQ, Chandra A, Phillips DB, Day SW, Borkholder DA (2015). Pulse wave velocity prediction and compliance assessment in elastic arterial segments. Cardiovasc Eng Technol.

[34] Lillie JS, Liberson AS, Borkholder DA (2016). Quantification of hemodynamic pulse wave velocity based on a thick wall multi-layer model for blood vessels. JFFHMT.

[35] Gribbin B, Steptoe A, Sleight P (1976). Pulse wave velocity as a measure of blood pressure change. Psychophysiology.

[36] Martin SL, Carek AM, Kim C, Ashouri H, Inan OT, Hahn J, Mukkamala R (2016). Weighing scale-based pulse transit time is a superior marker of blood pressure than conventional pulse arrival time. Sci Rep.

[37] Zhang G, Gao M, Xu D, Olivier NB, Mukkamala R (2011). Pulse arrival time is not an adequate surrogate for pulse transit time as a marker of blood pressure. J Appl Physiol (1985).

[38] Pinheiro E (2009). Pulse arrival time and ballistocardiogram application to blood pressure variability estimation.

[39] Kim C, Carek AM, Mukkamala R, Inan OT, Hahn J (2015). Ballistocardiogram as proximal timing reference for pulse transit time measurement: potential for cuffless blood pressure monitoring. IEEE Trans Biomed Eng.

[40] American Heart Association.

[41] McEniery CM, Cockcroft JR, Roman MJ, Franklin SS, Wilkinson IB (2014). Central blood pressure: current evidence and clinical importance. Eur Heart J.

[42] White W B, Berson A S, Robbins C, Jamieson M J, Prisant L M, Roccella E, Sheps S G (1993). National standard for measurement of resting and ambulatory blood pressures with automated sphygmomanometers. Hypertension.

[43] Espersen K, Jensen EW, Rosenborg D, Thomsen JK, Eliasen K, Olsen NV, Kanstrup IL (1995). Comparison of cardiac output measurement techniques: thermodilution, Doppler, CO2-rebreathing and the direct Fick method. Acta Anaesthesiol Scand.

[44] Shahgaldi K, Manouras A, Brodin L, Winter R (2010). Direct measurement of left ventricular outflow tract area using three-dimensional echocardiography in biplane mode improves accuracy of stroke volume assessment. Echocardiography.

[45] Hundley WG, Li HF, Hillis LD, Meshack BM, Lange RA, Willard JE, Landau C, Peshock RM (1995). Quantitation of cardiac output with velocity-encoded, phase-difference magnetic resonance imaging. Am J Cardiol.

[46] (2013). Center for Devices and Radiological Health, Food and Drug Administration.

